# Can Three-Dimensional Multiple Object Tracking Training Be Used to Improve Simulated Driving Performance? A Pilot Study in Young and Older Adults

**DOI:** 10.1007/s41465-023-00260-3

**Published:** 2023-04-24

**Authors:** Jesse Michaels, Romain Chaumillon, Sergio Mejia-Romero, Delphine Bernardin, Jocelyn Faubert

**Affiliations:** 1 Faubert Laboratory, School of Optometry, Université de Montréal, Montréal, Québec Canada; 2 Essilor International, Research and Development Department, Paris, France; 3 Essilor Canada, Saint-Laurent, Canada

**Keywords:** Multiple object tracking (MOT), Driving, Cognitive training, Speed-of-processing, Attention

## Abstract

Driving ability has been shown to be dependent on perceptual-cognitive abilities such as visual attention and speed of processing. There is mixed evidence suggesting that training these abilities may improve aspects of driving performance. This preliminary study investigated the feasibility of training three-dimensional multiple object tracking (3D-MOT)—a dynamic, speeded tracking task soliciting selective, sustained and divided attention as well as speed of processing—to improve measures of simulated driving performance in older and younger adults. A sample of 20 young adults (23–33 years old) and 14 older adults (65–76 years old) were randomly assigned to either a 3D-MOT training group or an active control group trained on a perceptual discrimination task as well as *2048.* Participants were tested on a driving scenario with skill-testing events previously identified as optimal for cross-sectional comparisons of driving ability. Results replicated previously identified differences in driving behaviour between age groups. A possible trend was observed for the 3D-MOT trained group, especially younger adults, to increase the distance at which they applied their maximum amount of braking in response to dangerous events. This measure was associated with less extreme braking during events, implying that these drivers may have been making more controlled stops. Limitations of sample size and task realism notwithstanding, the present experiment offers preliminary evidence that 3D-MOT training might transfer to driving performance through quicker detection of or reaction to dangerous events and provides a rationale for replication with a larger sample size.

## Introduction

Driving is undoubtedly a highly complex task. While experienced drivers can often be fooled by the relative ease with which they control their vehicles, this performance is subserved by a panoply of sensory, motor and cognitive systems working in concert (Eby et al., 2008). Indeed, research has now come to emphasize the importance of perceptual-cognitive abilities such as visual attention, visuospatial skills and speed of processing above purely visual sensory measures in relation to driver safety (Anderson et al., 2005; Ball et al., 1993; Owsley & McGwin, 1999, 2010; Wood, 2002). Such research is typically conducted in older adult populations due to their well-established declines in components of attention (Commodari & Guarnera, 2008; Lawrence et al., 2018; Salthouse et al., 1984) and speed of processing (Eckert et al., 2010; Salthouse, 1996). As discussed by Zicat et al. (Zicat et al., 2018), this relationship between safety and perceptual-cognitive ability has often been neglected in research on younger drivers; they found that such abilities accounted for driving performance even in young adults and even while accounting for personality traits and driving attitudes. Similarly, Backs et al. (Backs et al., 2011) also found that attention explained driving performance variance in a large sample of varying age groups. Nonetheless, studying the ageing driver context is crucial due to the ongoing demographic shift occurring in industrialized countries (Population Division, 2019) and findings suggesting that some older adults may be at an elevated risk of accidents relative to middle-aged drivers (Braver & Trempel, 2004; McGwin & Brown, 1999; Ryan et al., 1998).

An impressive body of literature on the Useful Field of View (UFOV)—a computerized measure of selective and divided attention as well as speed of processing—demonstrates that deficits in these functions are predictive of long-term negative driving outcomes such as driving errors (Anstey & Wood, 2011), crash involvement (Ball et al., 2006; Owsley et al., 1998; Rubin et al., 2007) and eventual driving cessation (Edwards et al., 2008, 2010; Kokkinakis et al., 2021; Ross et al., 2017). UFOV and other measures of decreased speed of processing have also been linked to increased driver self-regulation (Baldock et al., 2006; Roenker & Joyce, 2009) and driving errors (Aksan et al., 2015). Additionally, research demonstrates that UFOV is capable of predicting simulated and on-road driving performance in experimental studies (Hoffman et al., 2005; Wood et al., 2012). While predicting differences in driving performance and outcomes is already an impressive feat, some research has suggested that training aimed at improving UFOV may actually enhance driver safety. Roenker et al. (Roenker et al., 2003) showed that speed-of-processing training resulted in improved driving performance in a simulator and decreased reaction times. A longitudinal study by Ross et al. (Ross et al., 2017) found decreased driving cessation among individuals at-risk for future mobility declines following training and booster sessions. Such results are not consistent, however, as both Gaspar et al. (Gaspar et al., 2012) and Tsotsos et al. (Tsotsos et al., 2010) did not observe any improvements related to training. This is perhaps unsurprising considering how complex a task driving is and the multitude of methods one could use to quantify its performance. That said, the great social value of maintaining road safety for all offers a clear impetus to continue evaluating whether such training can indeed transfer to measures of driving performance.

Recently, we and two other groups of researchers demonstrated that attentional ability and speed of processing measured by three-dimensional multiple object tracking (3D-MOT) could also predict measures of simulated driving performance (Bowers et al., 2011; Michaels et al., 2017; Woods-Fry et al., 2017). The version of the task used—which assesses the speed at which individuals can simultaneously track and attend to multiple moving objects amidst identical distractors—was implemented using commercially-available technology known as NeuroTracker™. It has a number of differences compared to the UFOV: most notably, it assesses dynamic attention unlike the static stimuli of the UFOV. Additionally, its difficulty is more adaptable to a wide range of populations and individual baselines (Faubert, 2013).

3D-MOT training has been shown to enhance young adult selective and distributed attention, visual information processing and working memory function measured through neuropsychological tests and changes in associated quantitative electroencephalographic activity (Parsons et al., 2016). It has also been shown to transfer to UFOV performance in young and middle-aged adults (Harenberg et al., 2021; Michaels et al., 2022), improve passing decision-making accuracy in soccer players (Romeas et al., 2016), boost working memory span in military populations (Vartanian et al., 2016) and improve attention in students with neurodevelopmental conditions (Tullo et al., 2018). However, unlike with UFOV training, no study has ever evaluated possible transfer to driving performance following this training paradigm. Thus, we decided to investigate whether 3D-MOT training could produce a measurable effect on the performance of a simulated driving task.

## Materials and Methods

### Participants

**Fig. 1:**
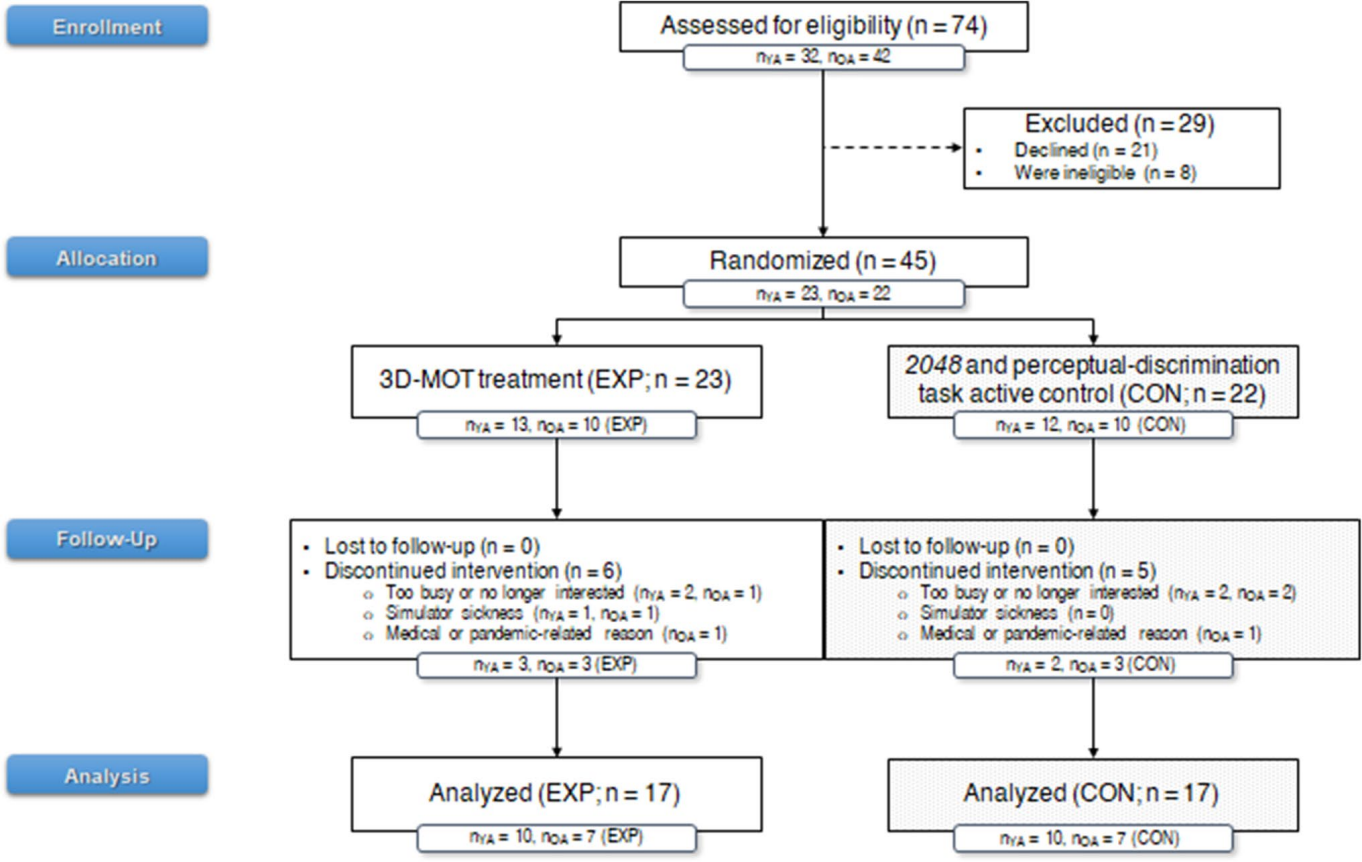
Flow diagram outlining participant inclusion and randomization process. Sample size information about young adult (YA) and older adult (OA) and their distribution in experimental (EXP) and active control (CON) treatments is provided for each step

To investigate training outcomes as well as the potential effect of age on training outcomes, we assessed 32 young adults (YA) and 44 older adults (OA) for eligibility. As shown in Fig. 1, if a participant was found to be eligible, they were randomized into either an experimental (EXP; n_EXP_ = 23) or active control group (CON; n_CON_ = 22) via a computer randomization script. As a result of the global COVID-19 pandemic necessitating early termination of the study, our final sample consisted of 20 young adults (YA) that were 23–33 years old and 14 older adults (OA) that were 65–76 years old (*N* = 34; N_YA_ = 20, N_OA_ = 14) distributed in equal quantity to experimental and active control groups (n_EXP_ = 17, n_CON_ = 17). The young adult experimental and active control groups were statistically similar in age (M_YA_ ± SD = 27.5 ± 3.21 vs. 29.1 ± 2.77), sex ratio (n_YAfemale_ = 5 vs. 4) and years licensed to drive (M_YA_ ± SD = 8.6 ± 3.6 vs. 9.8 ± 4.34). The same was true for older adult age (M_OA_ ± SD = 70.43 ± 3.69 vs. 68.0 ± 3.37), sex ratio (n_OAfemale_ = 3 vs. 2) and years licensed (M_OA_ ± SD = 53.43 ± 5.09 vs. 52.57 ± 4.12). Regardless of randomization, participants were told that they were recruited to test the effectiveness of a computer-based perceptual-cognitive training programme.

All participants had normal or corrected-to-normal vision, consisting of visual acuity score of 6/7.5 or better with both eyes using an ETDRS chart, stereoscopic acuity of 50 s of arc or better using the Randot test and a normal visual field using a Humphrey visual field analyser. Additionally, all participants had a normal score (≥ 26/30) on the Montreal Cognitive Assessment (MoCA) suggesting no mild cognitive impairment. They were free of visual, sensory, motor and neurological impairments as well as any diagnosis of neurodevelopmental disorders, and all possessed a valid driver’s licence for a minimum of 5 years. The study adhered to the tenets of the Declaration of Helsinki (last modified, 2013), and all tests and procedures were approved by the ethics committee of the Université de Montréal (CERES; Comité d’éthique de la recherche en santé certificate n° 16–130-CERES-D). All volunteers signed forms indicating informed consent and received a compensation of $15 at the end of each session.

### Outcome Measures

#### Apparatus

To evaluate participants’ driving performance when faced with dangerous situations, a VS500M car driving simulator (Virage Simulation Inc.®) was used for all driving sessions. It is a high-fidelity motion platform driving simulator that uses real car parts in the cockpit, including a seat, force feedback steering wheel, dashboard, controls, indicators, automatic transmission as well as accelerator and brake pedals. Three 1280 × 720 pixel 50-inch plasma screens provided a 180° front field of view while two smaller screens placed laterally and behind the cockpit replicated the car’s blind spots. Rear-view and side mirrors were digitally rendered in the front screens to approximate their physical locations in a real car. Additionally, motion and sound cues were used to enhance realism and immersion even further. The driving cabin was mounted on a three-axis platform with electric actuators that recreated the haptic feedback of acceleration, braking, engine vibration and road texture as a function of driving speed. Naturalistic engine and surrounding road sounds were recreated via a stereo sound system and Doppler shifts were applied to the sounds of passing traffic as a function of driving speed.

#### Scenario and Driving Measures

Driving simulator validity is highly dependent on proper scenario and driving measure selection and this is especially true in the context of cross-sectional research (Blickensderfer et al., 2005; Matas et al., 2016; Mullen et al., 2011). Thus, we elected to reuse the same rural scenario and driving measures previously described in our previous large-scale methodological study (Michaels et al., 2017). Compared to the alternatives, the rural scenario was shown to be the most sensitive to well-described age differences in driving performance as well as the best at eliciting realistic driving behaviours. This scenario was designed to be of moderate complexity and mental workload, following the work of Paxion et al. (Paxion et al., 2014) analyzing driving situation complexity and mental workload in terms of different road designs, layouts and traffic densities. It contained sections of road with three different speed limits: 90, 70 and 50 km/h. Participants were instructed to drive as naturally as possible while respecting the posted speed limits, road signage and other drivers, and to follow visual and auditory navigational instructions provided by the scenario.

To reduce previously identified mean driving speed variability between older and younger adults while still allowing participants full control over the vehicle, auditory feedback was provided to participants if their driving speed surpassed or fell below the posted speed limits by more than 5 km/h. This feedback took the form of unobtrusive high- and low-pitch tones (for above and below the speed limit, respectively) that obviated the need for participants to shift their gaze from the road. Participants were instructed to use these cues and the three different posted speed limits to maintain a reasonable driving speed except in situations where they needed to respond to on-road situations. To have a large enough sample of each participant’s driving behaviour, performance was averaged across seven skill-testing events that were triggered at pre-programmed locations along the route. These events required evasive manoeuvres or sudden braking to navigate safely without collisions. The scenario included both single-phased (i.e. the hazard is always visible) and two-phased materialized hazards (i.e. the hazard is hidden before becoming visible) following the event typology described by Borowsky and Oron-Gilad (Borowsky & Oron-gilad, 2013). Two variants of this scenario were programmed with the location of events shuffled around to reduce any learning effects from pre to post. Presentation of each variant in either pre- or post-training was randomized and counterbalanced across subjects to control for any unintended bias in task difficulty.

As summarized in Table 1, a total of nine measures were previously identified as pertinent and non-redundant descriptors of driving performance in the rural scenario (Michaels et al., 2017). Additionally, correlation and multiple linear regression analysis demonstrated links between 3D-MOT performance and a number of these measures. Thus, we hypothesized that if perceptual-cognitive training can measurably improve driving safety, it would be most evident through these measures.

**Table 1:** Definition of the most pertinent measures identified by Michaels et al. (2017) and the units in which they were recorded

	Measure	Unit	Description
1	Crash	*n*	Whether a collision occurred or not during the event
2	Near Crash	*n*	When within an event: • Subject brakes harder than a given threshold while driving at a speed greater than 5 m/s (18 km/h) • The steering wheel is turned more than 60° while driving faster than a speed threshold (5 m/s) • The participant drives within 3 m of an object while travelling at a speed greater than 10 m/s (36 km/h)
3	Mean Speed	*km/h*	Average speed of all driving. Data points where speed was inferior to 10 km/h or recorded 300 m before and 100 m after an event were discarded from the averaging
4	SDLP	*m*	Standard deviation of lateral position. Identical exclusion criteria as mean driving speed were applied. Additionally, for each data point, lateral positions recorded 10 s before and after a lane change were excluded from the averaging
5	Max Brake	*n*	Hardest amount of braking applied during event of interest. Ranges between 0 (= no braking applied) and 1 (= brake pedal is fully depressed)
6	Distance at Max Brake	*m*	Distance from event of interest at which “Max brake” is recorded
7	Max Steer Change Rate	*°/s*	Most extreme (in terms of range and speed) left or right steering wheel position change during event of interest
8	Distance at Max Steer Change Rate	*m*	Distance at which “Max steer change rate” is recorded during event of interest
9	Steer Range	*°*	Difference in degrees between leftmost and rightmost steering wheel position for event of interest

*n*, undefined unity

#### Attention and Speed of Processing: the UFOV Test

As previously discussed, research has demonstrated significant correlation between UFOV and MOT ability (Bowers et al., 2011). It has been previously remarked that performance of 3D-MOT and UFOV is likely subserved by common cognitive processes such as divided and selective attention as well as speed-of-processing (Michaels et al., 2022). Considering the established literature demonstrating the utility of UFOV as a predictor of driving performance and the evidence that it may improve driver reaction time, we elected to include the UFOV version 7 as an additional mid-level transfer outcome measure of 3D-MOT training. This version of the test (described in in detail by Woutersen et al. (2018)) is composed of three subtests that measure, respectively, (1) processing speed, (2) processing speed under a divided attention condition and, finally, (3) processing speed under a selective attention condition. To date, no study has demonstrated transfer of 3D-MOT training to UFOV performance in healthy older adults.

### Protocol

The study was divided into three phases occurring over a period of 7 weeks: (1) the pre-training phase (week 1), (2) the training phase (weeks 2–6) and (3) the post-training phase (week 7).

The first of these phases was identical for all subjects and consisted of two in-person sessions separated by a minimum of 2 days. The first of these consisted of a visual exam including the ETDRS (Early Treatment Diabetic Retinopathy Study) visual acuity test, Humphrey visual field test and the Randot stereoacuity test meant to screen any drivers with uncorrected visual deficits. Additionally, participants were screened for mild cognitive impairment using the MoCA. They were interviewed to confirm that they were never diagnosed with neurodevelopmental disorders or untreated health problems affecting their equilibrium or heart. All were free from neurodegenerative diseases and diabetes and were not routinely taking any medications that could affect their vigilance or attention. Finally, all participants confirmed that they had never previously participated in any research studies on driving and that they held a driver’s licence for a minimum of 5 years. Following the screening step, participants’ visual processing speed was assessed using the UFOV. At the end of this session, participants all tried the driving simulator in an unrecorded 12-min driving session consisting of two 6-min-long highway driving scenarios without skill-testing events. This initial introduction was done to allow participants to become familiar with the handling of the vehicle before testing sessions and because it has been shown to reduce the effects of Simulator Adaptation Sickness (Teasdale et al., 2009). The second pre-training session included an assessment of baseline 3D-MOT tracking speed and perceptual discrimination thresholds. As all participants were naïve to both tasks, they were first read instructions by the experimenter and given six practice trials of 3D-MOT prior to the actual assessment to make sure they understood the instructions. Following these tests, participants were tested on the driving simulator. The presence and severity of simulator sickness symptoms were measured before and after each driving test via the Simulator Sickness Questionnaire (SSQ) and change scores were computed to determine the effects of the simulator (Kennedy et al., 2009).

During the second phase, all participants were required to travel to the laboratory for ten 30-min, single-blind training sessions scheduled twice per week for five consecutive weeks (5 h total). This number of sessions and session duration was selected as it has been previously used to successfully demonstrate transfer in young adults (Romeas et al., 2016). As previously described, the experimental group’s training sessions consisted of three series of twenty 3D-MOT trials while the active control group underwent an alternate training of three series of a visual discrimination task followed by 15 min of *2048*. The experimenter read the rules of *2048* to participants in the active control group at the first training session and demonstrated how to use the keyboard arrows to control the movement of the tiles until participants clearly understood the control scheme and goal of the game.

The post-training phase consisted of a final two sessions mirroring the pre-training phase. The first of these included post-training 3D-MOT and perceptual discrimination measures and the second was dedicated to post-training driving simulator assessment using whichever variant of the rural scenario was not previously assigned at the pre-training driving test.

#### Three-Dimensional Multiple Object Tracking (EXP; Experimental Group)

**Fig. 2:**
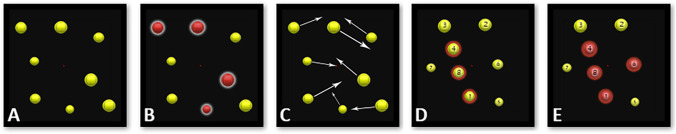
The five phases of a 3-dimensional multiple object tracking trial: **A** Eight identical spheres are randomly positioned in a virtual 3D cube. **B** The four target spheres are identified by becoming temporarily highlighted. **C** The target spheres revert to their standard appearance and all spheres begin randomly moving along linear paths within the cube. **D** The spheres stop moving and gain numerical labels to allow participants to identify the target spheres. **E** The correct spheres are highlighted and additional auditory feedback is provided

The 3D-MOT task requires simultaneous tracking of four randomly moving, dynamically interacting spherical targets while simultaneously ignoring four identical distractors for a continuous 8 s. The stimuli were displayed on a 65-in. Panasonic 3D TV screen. Subjects wore Panasonic active shutter 3D glasses while being seated on a chair placed 150 cm from the screen. As depicted in Fig. 2, each trial can be broken down into five phases. If a participant was able to successfully track and identify all four targets, then the trial was registered as successful and the movement speed of the stimuli in the following trial increased. Otherwise, stimuli speed was decreased. These changes followed a 1-up 1-down adaptive staircase protocol (Levitt, 1971) that varied speeds more greatly for early inversions than later ones in order to quickly identify the optimal speeds to train each participant. Correct or incorrect responses on each trial resulted in a proportional speed increase or decrease of 0.05 log units, respectively. Performance on the task was defined by a final tracking speed threshold computed based on the last four reversals of the staircase. Participants completed three series of 20 trials and the three tracking speed thresholds were subsequently averaged and log transformed to have a final value for each session.

#### Perceptual Discrimination and 2048 (CON; Active Control Group)

**Fig. 3:**
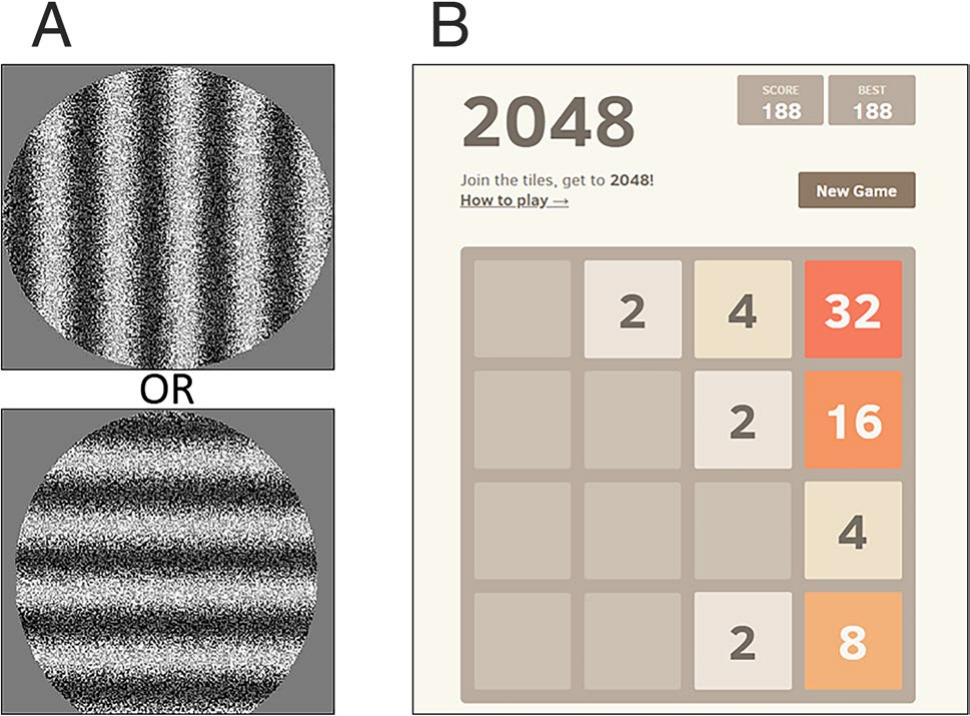
The two training tasks used in the active control group. **A** Examples of the stationary vertical and horizontal Gabor patches presented in the orientation perceptual-discrimination task before any modulation of contrast. Participants were forced to choose whether each stimulus presented was oriented either vertically or horizontally and correct answers resulted in subsequent presentations having decreased Michelson contrast and vice versa. **B** The 4 × 4 game grid and example tiles for *2048*. Players interact with the game solely via the four arrow keys to slide all the current game tiles in the chosen direction and then a new 2 or 4 tile is randomly added to the grid. When two identically numbered tiles collide, they combine to form the next highest factor of 2048, and the player’s score is increased by the value of that tile. The goal of the game is to create a 2048 tile and/or to achieve the highest possible score before the entire grid fills up in a configuration that blocks any further moves

As shown in Fig. 3, participants in the active control group all practised two different tasks. The first of these tasks used for the alternate training was a simple first-order (i.e. luminance-defined), forced-choice orientation discrimination task using sine-wave gratings (see (Legault, 2012) for a description). Using stimuli like those shown in Fig. 3A, participants were required to identify if the sine-wave grating was oriented either vertically or horizontally by pressing the up-arrow key or right arrow key, respectively. They were provided with auditory feedback indicating whether their response was correct or not after each trial. The gratings’ luminance was modulated following a 2-up 1-down staircase procedure and the minimum contrast threshold for stimulus orientation discrimination was estimated from the last six reversals of the staircase. This task was selected for its potential to demonstrate low-level perceptual learning without expected transfer to higher-level cognitive functions solicited by 3D-MOT.

Following three series of the discrimination task, the last remaining 15 min in each 30-min training session was spent by having active control participants play the challenging open-source puzzle game *2048* (https://play2048.com/) shown in Fig. 3B*.* Its use as an active control task for 3D-MOT has previously been described in the literature (Tullo et al., 2018). Additionally, the easy-to-grasp rules and simple control scheme makes the task particularly well-suited for older and younger adults alike. Once a participant could make no more valid moves (i.e. they had a “game-over”), their score was noted to track their progress. The highest score achieved was used for analyses if a participant played multiple rounds in a single session. The display and seating distance for both active control tasks were identical to the experimental task.

### Statistical Analysis

We first conducted preliminary analysis on the intervention and outcome measures to check the data distributions and to examine if baseline results were consistent with past observations. Two extreme outliers were detected (n_YA_CON_ = 1, n_OA_EXP_ = 1) using boxplots of pre-training and post-training perceptual discrimination scores (following (Tabachnick & Fidell, 2013)). These data points were removed from subsequent analysis involving that measure due to the fact that the participants’ visual examination and reported medical history strongly precluded the possibility of such severe perceptual deficits, instead suggesting they may have misunderstood the task. As the UFOV task used in this study consists of 3 subtests, a composite score was calculated across subtests 2 and 3 (following (Ball et al., 2013) due to the fact that over 90% of participants achieved the best possible performance for the first subtest. Three outliers were detected (n_YA_CON_ = 1, n_OA_EXP_ = 1, n_OA_CON_ = 1) for pre-training UFOV scores but only the data for the young adult participant was removed from subsequent analyses due to the expected heterogeneity of older adult UFOV performance.

Consistent with our past findings (Michaels et al., 2017), there was a notable difference in the naturally adopted mean speeds of older (M_OA_ ± SD = 62.05 ± 4.75) and younger adults (M_YA_ ± SD = 68.09 ± 4.43) during the first exposure to the rural scenario. The 9.73% difference in mean speeds between older and younger adults, while extremely similar to the 10.33–12% difference we previously described, also shows considerably decreased variability compared to those data. This may suggest that the auditory feedback we used to try and help participants regulate their speeds was effective at stabilizing speeds between individuals even if it could not entirely compensate for older adults’ propensity to adopt naturally slower mean driving speeds.

In order to shed light on how the various driving measures were related with one another and with age, correlations were conducted both on pre-training and post-training data. Pearson partial correlations controlling for mean speed were computed to account for the fact that age still appeared to influence naturally adopted mean driving speed despite the auditory feedback. Spearman correlations were performed instead for measures that did not follow a normal distribution.

To compare training task, UFOV, SSQ and driving performance metrics between both groups, multiple linear mixed-effects models with repeated measures design were constructed including *Group* (i.e. Exp vs. Con) and *Age* (i.e. YA vs. OA) as categorical predictors and *Session* (i.e. Pre vs. Pos) as a within-subjects factor. *Group* was omitted as a factor when analyzing *2048* data due to the fact that the experimental group never performed the task. Generalized linear mixed models were used instead for variables not following a normal distribution. This approach is the most widely recommended method of analyzing pretest–posttest data (Bolker et al., 2009; O’Connell et al., 2018). Finally, we analysed SSQ change scores following the driving test in both pre-training and post-training sessions to rule out the possibility that one treatment group might have experienced more potentially confounding simulator sickness symptoms purely by chance.

## Results

### Pre-training Partial Correlation Analysis

**Fig. 4:**
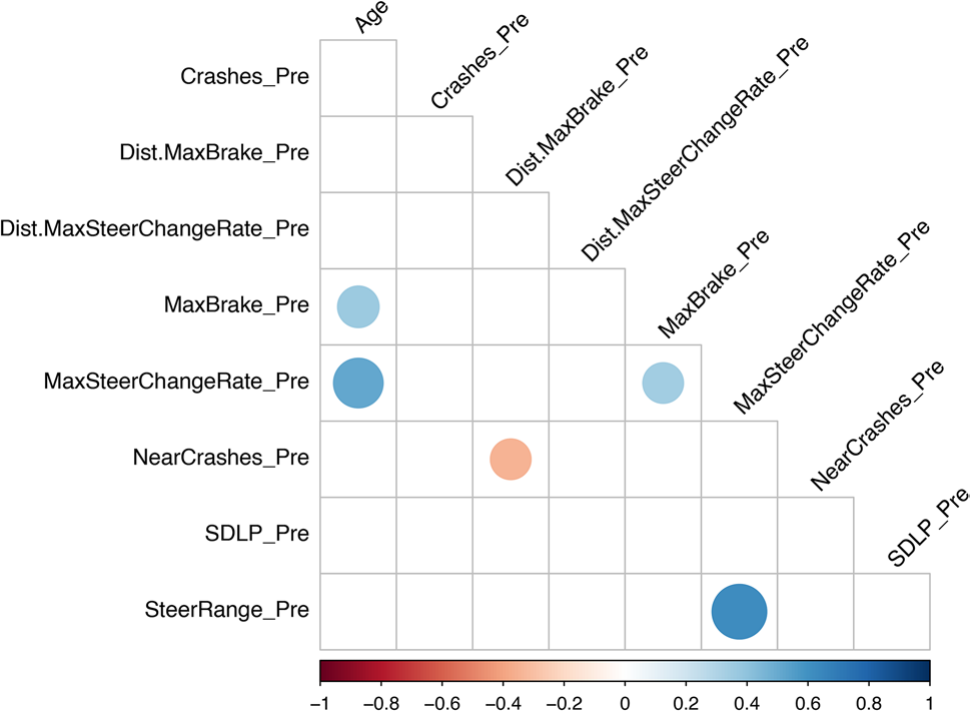
Graphical representation of the pre-training partial correlation analysis controlling for mean speed generated in the R statistical environment (R Core Team, 2022). Only significant correlations (*p* < .05) appear on the figure. The size of each circle represents the magnitude of the correlation and the colour represents the direction

A graphical representation of the results of the pre-training partial correlation analysis on driving measures can be found in Fig. 4. It was found that, after controlling for mean speed, ‘Max Steer Change Rate’ correlated positively with both ‘Max Brake’ [*r*(31) = 0.35,* p* = 0.048] and ‘Steer Range’ [*r*(31) = 0.63, *p* < 0.001]. Of these three measures, ‘Max Brake’ [*r*(31) = 0.36, *p* = 0.038] and ‘Max Steer Change Rate’ [*r*(31) = 0.52, *p* = 0.002) correlated positively with age. These finding are consistent with our previous research on these measures and are reflective of the compensatory actions taken by drivers in response to dangerous events (Michaels et al., 2017). Older adults appeared to make more abrupt, less smooth driving manoeuvres on both the steering wheel and the brake pedal in response to dangerous events—possibly reflecting a compensatory mechanism resulting from slowed information processing speed. A significantly negative correlation between ‘Distance at Max Brake’ and ‘Near Crashes’ [*r*(31) =  − 0.37, *p* = 0.033] also suggests that participants responding to events earlier were less likely to get into near crashes.

Finally, a partial correlation was computed to study the relationship between baseline 3D-MOT and UFOV. Considering that performance on both measures is known to decrease with age, we elected to control for this variability inherent in our sample to better understand the association between the two measures. Consistent with other research (Bowers et al., 2013), a moderate-strength negative correlation was detected [*r*(30) =  − 0.50, *p* = 0.004]. This further reinforces the idea 3D-MOT tests similar aspects of cognitive function as the UFOV but that performance on the task is partially subserved by other cognitive abilities.

### Post-training Analyses

#### Partial Correlations

**Fig. 5:**
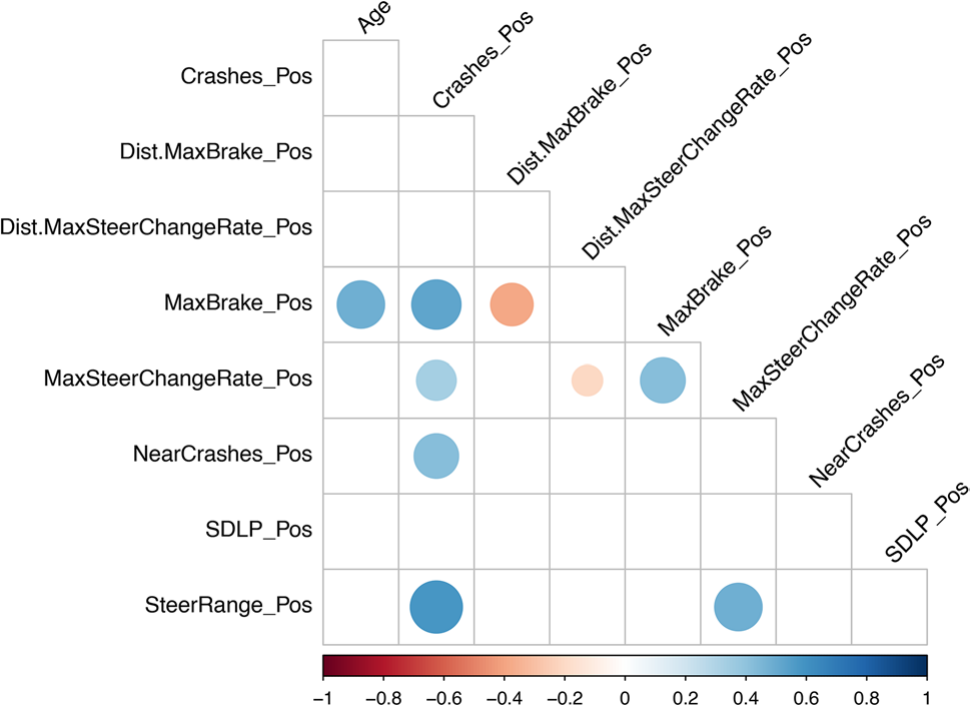
Graphical representation of the post-training partial correlation analysis controlling for mean speed. Only significant correlations (*p* < .05) appear on the figure. The size of each circle represents the magnitude of the correlation and the colour represents the direction

The post-training partial correlation analysis can be found represented in Fig. 5. Here, ‘Max Brake’ was positively correlated with ‘Crashes’ [*r*(31) = 0.50, *p* = 0.003], ‘Steer Range’ [*r*(31) = 0.60, *p* < 0.001] and continued to be correlated with ‘Max Steer Change Rate’ [*r*(31) = 0.43, *p* = 0.013] as well as Age [r(31) = 0.44, p = 0.01]. ‘Crashes’ were correlated with ‘Near Crashes’ [r(31) = 0.45, p = 0.008], ‘Steer Range’ [*r*(31) = 0.59, *p* < 0.001] and ‘Max Steer Change Rate’ [*r*(31) = 0.36, *p* = 0.039]. ‘Max Steer Change Rate’ was negatively correlated with ‘Distance at Max Steer Change Rate’ [*r*(31) =  − 0.36, p = 0.041] and continued to be positively correlated with ‘Steer Range’ [*r*(31) = 0.48, *p* = 0.004]. Finally, ‘Max Brake’ correlated negatively with ‘Distance at Max Brake’ [*r*(31) =  − 0.39, *p* = 0.024]. This last result suggests that individuals braking earlier were more likely to make controlled and deliberate stops (and vice versa), while the rest of these results paint a picture of individuals engaging in particularly extreme, last-minute driving manoeuvres when faced with an imminent and unanticipated risk of collision. Such results are coherent with behavioural reports by Pacaux-Lemoine et al. of drivers forced into similar circumstances (Pacaux-Lemoine et al., 2011). Additionally, these individuals may have been more likely to get into near crashes as well; a finding that could reflect a certain profile of riskier driver or that may imply that individuals with higher crash rates responded to events later in general.

### Mixed Models and t-Tests: Training Tasks

**Fig. 6:**
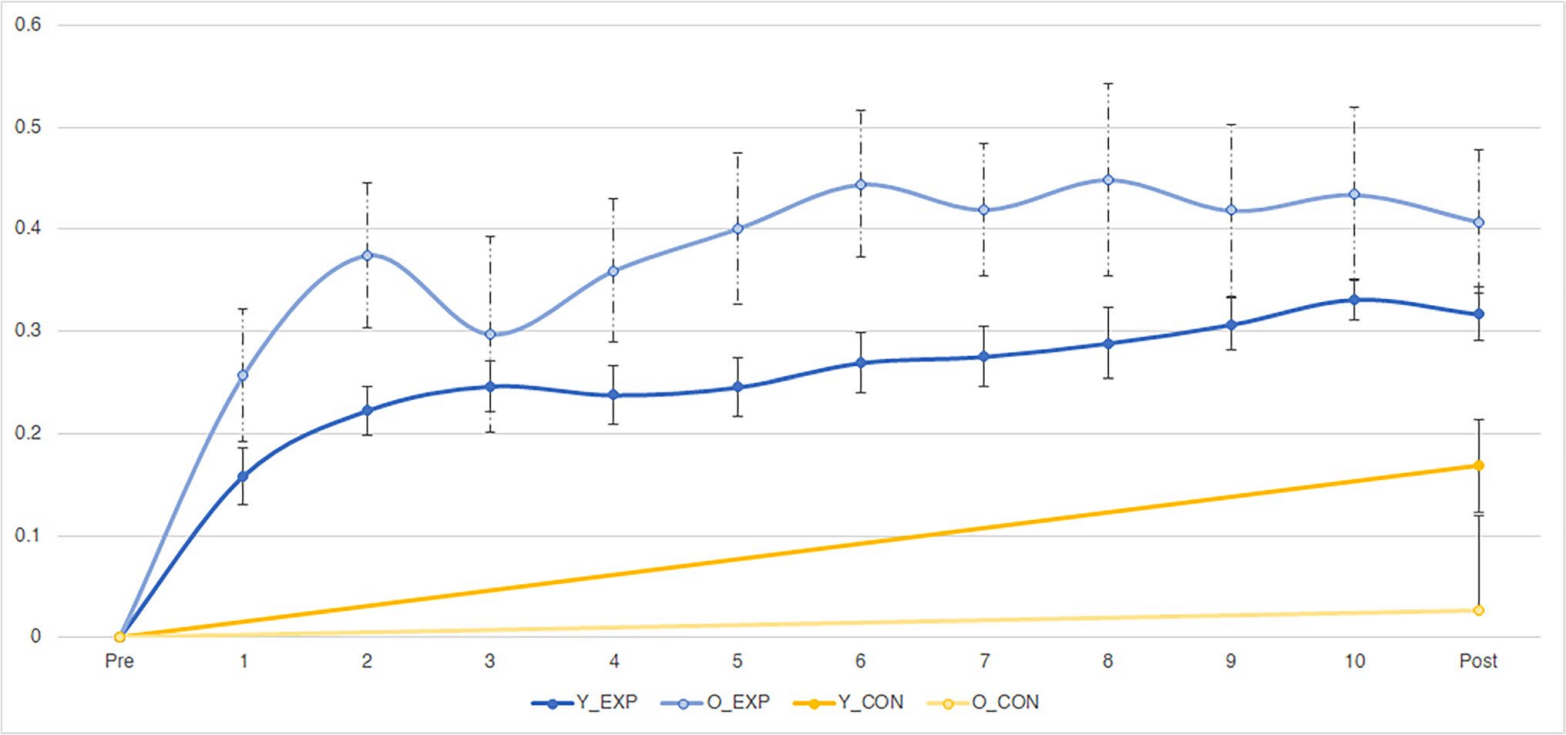
Normalized learning curves (session scores − initial score) with group means and standard error bars for 3D-MOT log-transformed scores. Note the appearance of a plateau around session 6 for the older adult experimental group (O_EXP) compared to the younger adult experimental group (Y_EXP)

Next, we examined outcomes from the training tasks. As shown in Table 2, 3D-MOT performance showed the expected main effect of Age [*F*(1,30) = 41.03, *p* < 0.001, η^2^_p_ = 0.58], an expected Group*Session interaction [*F*(1,30) = 37.80, *p* < 0.001, η^2^_p_ = 0.56] and a Group*Age*Session interaction [*F*(1,30) = 11.23,* p* = 0.002, η^2^_p_ = 0.27] driven by the fact that the young adults in the control group (but not older adults) also demonstrated some improvement on the task (see Fig. 6). Only a significant main effect of session (and no expected Group*Session interaction) was found for perceptual discrimination scores [*F*(1,28) = 5.36, *p* = 0.03, η^2^_p_ = 0.16]. Finally, a main effect of Session was observed for *2048* [*F*(1,4) =  − 23.92, *p* = 0.008, η^2^_p_ = 0.71], signifying that participants in the active control group improved at the task. The lack of a significant main effect of Age or an Age*Session interaction suggests that older and younger adults did not differ in terms of their baseline performance or improvement on the task.

**Table 2:** Summary of the mixed model analyses for training tasks

*Measure*	Group (1)	Age (2)	Session (3)	1*2	1*3	2*3	1*2*3	R^2^M	R^2^C
*3D-MOT*	0.448	** < 0.001**	** < 0.001**	0.29	** < 0.001**	0.72	**0.002**	0.63	0.91
*Perceptual discrimination*	0.98	0.70	**0.03**	0.36	0.71	0.65	0.16	0.11	0.24
*2048*	N.A	0.89	**0.008**	N.A	N.A	0.69	N.A	0.51	0.62

Linear mixed models with Group (Exp vs. Con), Age (YA vs. OA) and Session (Pre vs. Pos) as factors were constructed to investigate differences in training task performance. Each main effect is named and assigned a number. Interactions between factors are indicated by these numbers. The resulting *p*-values are provided and bolded when significant. The final two columns represent the marginal (R^2^M) and conditional (R^2^C) *R*-squared values for each model. Note that the *Group* factor was omitted from analyses involving *2048* as the experimental group never performed the task

**Table 3:** Descriptive statistics of pre-training and post-training measures

	EXP			CON		
*Measure*	Pre	Post	∆	Pre	Post	∆
*3D-MOT* ^A,S,G*S,G*A*S^	− 0.02 ± 0.14 − 0.57 ± 0.26	0.28 ± 0.10 − 0.09 ± 0.13	+ 0.30 + 0.48	− 0.07 ± 0.22 − 0.32 ± 0.27	0.10 ± 0.13 − 0.30 ± 0.28	+ 0.17 + 0.02
*Perceptual discrimination* ^**S**^	0.022 ± 0.01 0.026 ± 0.01	0.023 ± 0.01 0.019 ± 0.01	+ 0.001 − 0.007	0.020 ± 0.00 0.026 ± 0.01	0.017 ± 0.01 0.022 ± 0.01	− 0.003 − 0.004
*2048* ^**S**^	N.A N.A	N.A N.A	N.A N.A	2609.2 ± 529.8 1221.1 ± 797.1	5597.6 ± 2175.6 5760 ± 3020.7	+ 2988.4 + 4538.9
*UFOV* ^**A**^	75.35 ± 28.08 255.76 ± 89.07	57.09 ± 24.01 220.79 ± 85.13	− 18.26 − 34.97	90.12 ± 28.80 224.56 ± 162.71	85.91 ± 18.34 231.27 ± 242.70	− 4.21 + 6.71
*Crash*	0.80 ± 0.63 1.14 ± 0.69	0.40 ± 0.52 0.57 ± 0.53	− 0.40 − 0.57	0.70 ± 0.67 0.71 ± 0.49	0.60 ± 0.52 0.71 ± 0.76	− 0.10 N.D
*Near Crash*	0.80 ± 0.79 0.71 ± 0.95	0.40 ± 0.7 0.71 ± 0.76	− 0.40 N.D	0.50 ± 0.71 1.0 ± 1.0	0.80 ± 0.92 0.43 ± 0.53	+ 0.30 − 0.57
*Mean Speed* ^**A**^	68.24 ± 4.18 59.63 ± 2.12	69.17 ± 4.54 61.12 ± 1.57	+ 0.93 + 1.49	67.94 ± 5.53 64.48 ± 5.53	69.08 ± 3.5 66.35 ± 6.61	+ 1.14 + 1.87
*SDLP* ^G*A*S^	0.28 ± 0.05 0.27 ± 0.03	0.29 ± 0.04 0.29 ± 0.05	+ 0.01 + 0.02	0.28 + 0.05 0.31 ± 0.03	0.30 ± 0.06 0.29 ± 0.05	+ 0.02 − 0.02
*Max Brake* ^**A,S**^	0.64 ± 0.10 0.67 ± 0.11	0.49 ± 0.15 0.57 ± 0.18	− 0.15 − 0.10	0.65 ± 0.15 0.71 ± 0.12	0.51 ± 0.12 0.70 ± 0.15	− 0.14 − 0.07
*Dist. at Max Brake*	41.63 ± 9.98 43.94 ± 19.93	49.27 ± 7.79 46.57 ± 14.05	+ 7.64 + 2.63	42.76 ± 13.71 44.71 ± 9.95	43.27 ± 9.74 42.67 ± 10.66	+ 0.51 − 2.04
*Max Steer Change Rate* ^**A**^	293.28 ± 46.09 363.67 ± 44.65	314.51 ± 73.55 315.15 ± 36.24	+ 21.23 − 48.52	293.34 ± 50.10 334.71 ± 59.24	322.80 ± 67.24 358.24 ± 50.06	+ 29.46 + 23.29
*Dist. at Max Steer Change Rate* ^G*A*S^	35.79 ± 7.93 27.40 ± 11.06	36.71 ± 12.17 32.28 ± 12.18	+ 0.92 + 4.88	26.70 ± 10.01 32.32 ± 12.80	32.61 ± 8.45 24.17 ± 8.59	+ 5.91 − 8.15
*Steer Range*	66.04 ± 10.78 71.76 ± 11.59	72.94 ± 10.28 68.23 ± 10.55	+ 6.9 − 3.53	68.97 ± 10.19 74.55 ± 12.45	75.93 ± 17.75 76.63 ± 10.64	+ 6.96 + 2.08
*SSQ* ^**A**^	13.46 ± 6.76 19.23 ± 9.32	7.85 ± 4.71 29.92 ± 11.93	− 5.61 + 10.69	20.20 ± 7.73 40.61 ± 13.79	14.59 ± 7.34 25.11 ± 18.37	− 5.61 − 15.5

Means and standard deviations are provided for both training groups (EXP vs. CON) and both age groups (OA below YA in each cell) in their original units. Additionally, the difference (∆) between pre- and post-training values are provided (N.D. = no difference). Finally, superscript letters for each factor (^G^ = Group, ^A^ = Age, ^S^ = Session) are provided next to specific measure names to indicate when a significant main effect and/or interaction was detected for that measure

### Mixed Models: Outcome Measures

Pre- and post-training mean outcome measure and training task data can be found in Table 3. Mixed models were constructed to investigate whether either training protocol transferred to relevant driving measures or UFOV ability and whether age had any influence. Additionally, a mixed model was constructed to investigate whether simulator sickness varied between treatment and/or age groups.

Consistent with the known propensity of older adults to experience more simulator sickness (Keshavarz et al., 2018), a trend for a significant main effect of Age was detected for SSQ change scores [*F*(1,30) = 3.70, *p* = 0.06, η^2^_p_ = 0.11] as shown in Table 4. Otherwise, no potentially biasing group or interaction effects were noted.

**Table 4:** Summary of the mixed model analyses for driving data

Measure	Group (1)	Age (2)	Session (3)	1*2	1*3	2*3	1*2*3	R^2^M	R^2^C
*UFOV*	0.87	** < 0.001**	0.18	0.65	0.14	0.88	0.46	0.37	0.92
*Crash*	0.98	0.45	0.20	0.67	0.20	0.67	0.31	0.08	0.08
*Near Crash*	0.97	0.67	0.39	0.75	0.80	0.62	0.11	0.10	0.10
*SDLP*	0.36	0.84	0.10	0.74	0.59	0.21	**0.04**	0.06	0.73
*Max Brake*	0.22	0.0**3**	** < 0.001**	0.35	0.34	0.08	0.41	0.26	0.58
*Dist. at Max Brake*	0.61	0.95	0.19	0.91	0.08	0.26	0.71	0.04	0.71
*Max Steer Change Rate*	0.73	**0.03**	0.57	0.93	0.08	0.10	0.16	0.16	0.45
*Dist. at Max Steer Change Rate*	0.21	0.23	0.68	0.44	0.35	0.24	**0.04**	0.13	0.47
*Steer Range*	0.16	0.55	0.30	0.66	0.64	0.21	0.65	0.08	0.08
*Mean Speed*	0.07	** < 0.001**	0.12	**0.05**	0.86	0.71	0.96	0.37	0.60
*SSQ*	0.33	0.06	0.53	0.92	0.30	0.80	0.30	0.10	0.28

Linear mixed models with Group (Exp vs. Con), Age (YA vs. OA) and Session (Pre vs. Pos) as factors were constructed to investigate differences in training task performance, UFOV, driving performance and simulator sickness symptoms (SSQ). Generalized linear models were used instead in cases where variables did not follow a normal distribution. Each factor is named and assigned a number. Interactions between factors are indicated by these numbers. The resulting *p*-values are provided and bolded when significant. The final two columns represent the marginal (R^2^M) and conditional (R^2^C) *R*-squared values for each model

No significant transfer to UFOV was detected. Both young and older adults in the experimental treatment group exhibited greater improvement in post-training performance on the task relative to controls (∆_YA_EXP_ =  − 18.26 and ∆_OA_EXP_ =  − 34.97 vs. ∆_YA_CON_ =  − 4.21 and ∆_OA_CON_ =  + 6.71). While the improvement in young adults alone has been previously demonstrated to be statistically significant (Michaels et al., 2022), older adults exhibited far greater variability in UFOV outcomes. Indeed, the increase in mean UFOV detection speed threshold observed only in the older adult control group was primarily attributable to one of the previously identified outliers who exhibited a substantially worsened score at post-training. In order to help rule out the possibility that these data points were exerting undue leverage on the statistical analysis, we subsequently examined winsorized the scores of the previously identified older adult outliers. This equalized the variability between the two older adult groups (SE_OA_ = 19.1 vs. 19.1) and revealed that the older adults in the experimental group had worse baseline UFOV scores compared to their control counterparts (M_OA_ = 236.8 vs. 172.8). Including these modified values in the linear model resulted in a trend toward a significant Group*Age interaction (*p* = 0.05) driven by these random baseline differences in the context of a small older adult sample size. Outright removal of the outliers pushed this trend to become statistically significant (*p* < 0.01). Older adults in the experimental group still exhibited a greater improvement in their UFOV scores compared to control subjects, but the magnitude of this difference was so small as to be practically nonexistent—especially when considering their lower baseline (∆_OA_ =  − 16.0 vs. − 10.9).

A main effect of Age was found for ‘Max Brake’ [*F*(1,30) = 5.02, *p* = 0.03, η^2^_p_ = 0.14]. This result is attributable to an overall tendency for older adults to brake harder that was further emphasized by a sharper decrease in the maximum amount of braking applied by both experimental and active control younger adults (− 0.15 and − 0.14 respectively) compared to older adults (− 0.10 and − 0.01) during the post-training driving events. Interestingly, this difference was clearly strongest between young adults and the active control older adults in particular, but the difference was not great enough to evince a significant interaction. The significant main effect of Session for ‘Max Brake’ [*F*(1,30) = 15.76, *p* < 0.001, η^2^_p_ = 0.34] is also explained by this pattern of results, suggesting that participants were likely more familiar with piloting the driving simulator at the second session and may also be related to beginning brake manoeuvres earlier. The main effect of Age on ‘Max Steer Change Rate’ [*F*(1,30) = 5.38, *p* = 0.03, η^2^_p_ = 0.15] is further indicative of the more abrupt actions taken by older adults when faced with dangerous events. Both of these results are consistent with correlations between age and these measures found for the rural scenario in our previous study (Michaels et al., 2017). While older adults adopted a mean speed slightly closer to their younger counterparts post-training, the model confirmed that there was still a strong main effect of age group on ‘Mean Speed’ [*F*(1,30) = 20.37, *p* < 0.001, η^2^_p_ = 0.40]. A Group*Age interaction was found for this measure [*F*(1,30) = 4.28, *p* = 0.05, η^2^_p_ = 0.12] and is attributable to a tendency for older adults in the control group to drive slightly faster than their counterparts in the experimental group (M_EXPOA_ = 60.4 vs. M_CONOA_ = 65.4). Possibly related to this was the presence of a significant 3-way interaction for ‘Distance at Max Steer Change Rate’ [*F*(1,30) = 4.52, *p* = 0.04, η^2^_p_ = 0.13] where it was found that only older adults in the control group exhibited a decrease in this measure (i.e. later responding) (∆_EXPYA_ =  + 0.92, ∆_EXPOA_ =  + 4.88, ∆_CONOA_ =  + 5.91 vs. ∆_CONOA_ =  − 8.15) as well as a 3-way interaction for ‘SDLP’ [*F*(1,30) = 4.50, p = 0.04, η^2^_p_ = 0.13] where the same pattern was observed (∆_EXPYA_ =  + 0.01, ∆_EXPOA_ =  + 0.02, ∆_CONOA_ =  + 0.02 vs. ∆_CONOA_ =  − 0.02).

Two interesting trends were also noted and are worthy of exploration considering the study’s limited statistical power. First, a trend for a Group*Session interaction for ‘Max Steer Change Rate’ [*F*(1,30) = 3.24, *p* = 0.08, η^2^_p_ = 0.10]. This is likely explained by the decrease observed for older adults in the experimental group at post-training (∆_EXPOA_ =  − 48.52) that was not observed for any other subgroup. Such a result may be related to increases in their ‘Distance at Max Steer Change Rate’ (∆_EXPOA_ =  + 4.88) and ‘Distance at Max Brake’ (∆_EXPOA_ =  + 2.63), possibly reflecting the fact that their slower mean speed would have allowed them to respond slightly earlier. Finally, a trend for a Group*Session interaction was found for ‘Distance at Max Brake’ [*F*(1,30) = 3.23, *p* = 0.08, η^2^_p_ = 0.10].

**Fig. 7:**
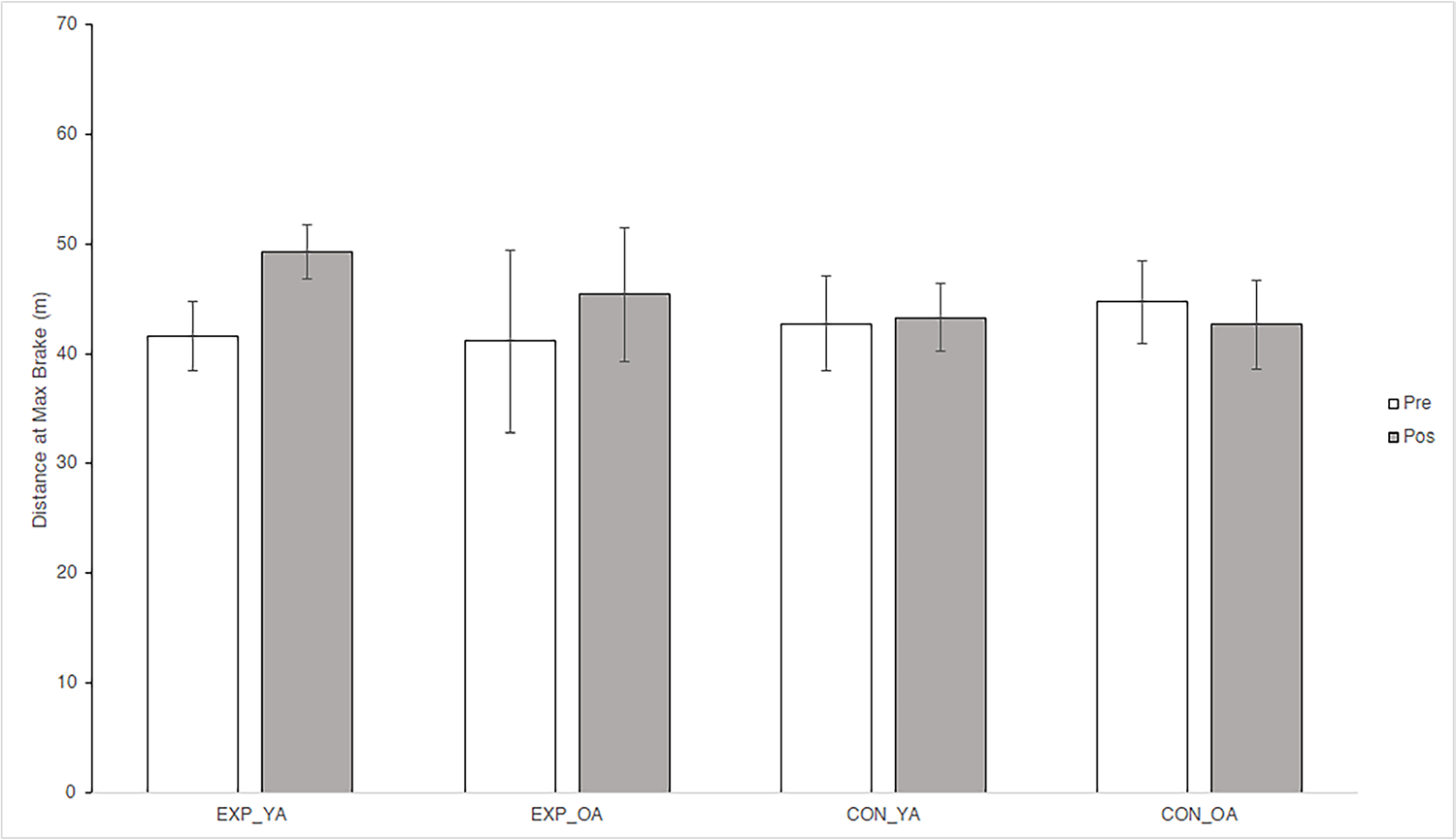
Pre- and post-training mean values for Distance at Max Brake separated by training and age group. Error bars represent standard error of the mean (SEM)

When comparing means of the two training groups (M_EXP_ ± SE = 47.92 ± 2.99 vs. M_CON_ ± SE = 43.0 ± 2.99), it appears that the group trained with 3D-MOT completed their braking manoeuvres slightly earlier when faced with dangerous situations. While the lack of a significant 3-way interaction would suggest that this improvement was not restricted to either young adults or older adults, the smaller difference and considerably greater variability in older adult post-training ‘Distance at Max Brake’ (shown in Fig. 7) implies that the effect was less widespread in that age group. It also suggests that this difference was not simply related to the slower mean driving speed observed for older adults in the experimental group. This finding is again consistent with speculation that the training paradigm employed in this study may have been less broadly successful in the older adult experimental group.

## Discussion

The main purpose of this study was to assess the feasibility of measuring changes in simulated driving performance following a 3D-MOT training protocol. Additionally, we also investigated whether the success of the protocol would vary as a function of age.

### Training Tasks

As expected, the experimental group showed significant improvement in 3D-MOT tracking speed thresholds as compared to the active control group. One interesting result to highlight is that of the improvement seen in the young adult active control group’s tracking thresholds even after only a baseline assessment (see Fig. 6). This result mirrors findings from Parsons et al. (Parsons et al., 2016) who also observed improvement in their young adult control group from pre- to post-testing session that was comparable to the learning exhibited by the experimental group at their first training session (i.e. their second exposure to 3D-MOT) and shows that learning of the task lasts at least 5 weeks. Notably, this learning did not appear to translate into any measurable transfer effect on the driving metrics. This is consistent with results from the ACTIVE study using UFOV training and meta-analysis of cognitive training programmes (Chiu et al., 2017) suggesting that at least 10 sessions of perceptual-cognitive training may be required to measure transfer over and beyond practice effects (Tabachnick & Fidell, 2013). An unexpected related finding was the observation that older adults in the active control group did not seem to maintain much, if any, learning by comparison to their younger control counterparts. This difference in the durability of this learning was strong enough to produce a statistically significant 3-way interaction for 3D-MOT scores and suggests that there is still much to be learned about how ageing affects consolidation of cognitive training and the optimal training protocols for different age groups.

Despite faster initial improvement, older adults in the experimental group appeared to demonstrate a plateau after their sixth training session and their thresholds also demonstrated much greater variability at all training sessions. This finding may be explained by our decision to use four targets during the 3D-MOT training task. Previous 3D-MOT research conducted with older and younger adults has instead often resorted to using only three targets. Legault et al. (Legault et al., 2013) showed the inverse—that is, a plateau for younger adults but not older adults—using this paradigm. The logic of choosing three as opposed to four targets is that older adults often exhibit degraded perceptual-cognitive ability that, in many cases, renders tracking four targets disproportionately more difficult relative to their younger counterparts. We elected to use four targets as we worried that the aforementioned plateauing shown in younger adults when trained with three targets might interfere with possible transfer here. Additionally, we wanted to keep the training parameters identical between subjects for ease of comparison. Instead, we may have inadvertently limited the learning potential of the older adult training group. Coupled with the small sample size of the present study and the limited number and duration of training sessions, these factors may have reduced our ability to detect even the type of mid-level transfer that is routinely showed in cognitive training studies. Future work that permits more granular stratification of older adult participants contingent on factors such as baseline performance may help shed light on such questions.

Similar differential transfer effects between younger and older adults have been reported in a training study by Dahlin et al. (Dahlin et al., 2008). Their results further demonstrated that initial age-related changes in the neural substrate solicited for performing the training task resulted in less overlap of brain activation with the untrained task for older adults. It is possible that similar age-related differences in neural activation could exist for the 3D-MOT task and, additionally, it is reasonable to assume that such differences would be magnified as the task’s complexity is increased via greater tracking load and speed. Considering how the adaptive nature of the 3D-MOT task is a key feature of its design, future training studies comparing younger and older adult outcomes should consider this trade-off carefully during experiment conceptualization. Future work could also benefit by following the recommendation of Lustig et al. (Lustig et al., 2009) to use neuroimaging data in order to provide a clearer mechanistic account of transfer following cognitive training.

The presence of a main effect of Session but a lack of a significant Group*Session interaction for the perceptual learning task suggests that both groups demonstrated rapid improvement at the task. That the experimental group also improved at the task is not inconsistent with the established literature on perceptual learning, which typically shows that it can occur rapidly (Gold & Watanabe, 2010). The lack of an interaction is somewhat surprising, however, considering the great difference in exposure to the task by the post-training test. It is possible that demonstrating stronger group-specific perceptual learning on such a task could require either more trials than was conducted here or that the study lacked adequate power to detect small additional differences following training. It is also possible that more obvious learning would have been demonstrated following a longer training duration. This is equally true for possible transfer to the driving task in the experimental group. Indeed, a recent meta-analysis suggests that cognitive training protocols using 24 or more sessions across 8 weeks produce significantly stronger effects than those with fewer sessions (Chiu et al., 2017). The fact that we were unable to demonstrate any age-related differences on the task is not particularly surprising. In fact, it has been suggested that the type of low-level processing required for perception of simple first-order stimuli may not be complex enough for age-related deficits to be consistently observed (Faubert, 2002). Finally, the learning effect demonstrated for *2048* suggests that active control participants were engaged in their training phase sessions.

### Driving Measures

Analyses on pre-training data replicated many of our past findings but not all of them. In particular, age was not positively correlated with ‘SDLP’ or ‘Crash’ and neither measure was significantly greater in the older adult group as was previously observed. This may either reflect factors unique to our sample or simply that the present study lacked adequate power to observe the same patterns. Indeed, while a major limitation of this study is the small sample size, it was especially limited for older adults who are already characterized by their greater heterogeneity in health and intervention outcomes (Ferrucci & Kuchel, 2021). What does appear to be consistent, however, is that age was associated with more extreme braking and steering manoeuvres. This was true even while older adults compensated for decreased reaction time by adopting slower mean driving speeds. These results are in line with and further reinforce the established literature on maladaptive or compensatory older adult driving behaviours (Feng et al., 2020; Fisher et al., 2011; Lee et al., 2003; Makishita & Matsunaga, 2008; Shanmugaratnam et al., 2010; Verhaegen et al., 1988). Finally, we showed that unobtrusive auditory feedback was capable of reducing some of the difference in naturally adopted mean speed between younger and older adults but could not eliminate it entirely.

As far as transfer to driving performance is concerned, the present results offer some evidence that 3D-MOT training may increase the distance at which drivers respond to dangerous events. Considering that such training has been shown to improve visual attention and speed of processing (Fleddermann et al., 2019; Michaels et al., 2022; Parsons et al., 2016; Tullo et al., 2018), this result could be the result of better distribution of attention or more efficient processing of the dynamic visual scene. (Cuenen et al., 2016) have highlighted attentional function as an important predictor of improved detection and reaction times during driving. Additionally, speed of visual information processing has for a long time been associated with driving safety and longitudinal driving outcomes through the body of UFOV research (Myers et al., 2000; Ross et al., 2017; Tabachnick & Fidell, 2013; Wood et al., 2012). (Mackenzie & Harris, 2017) also recently demonstrated important links between driving safety, attentional function as measured by MOT performance and visual speed of processing by way of differences in eye movement behaviours. Interestingly, our result has parallels with findings from Roenker et al. (Roenker et al., 2003) who showed that UFOV training improved drivers’ reaction times and suggested that this could translate to improved stopping times. Somewhat relatedly, studies with young action video gamers have also demonstrated improved perceptual speed-of-processing and reaction times observed across various tasks divorced from the context of gaming (Dye et al., 2009). However, considering the limitations of the current study, a replication with larger sample sizes would help assuage reasonable doubts about this interpretation.

Though an average relative gain of only about 5 m at the point of maximum braking seems modest, one should keep in mind that a driving speed of 70 km/h translates to roughly 19 m/s and 50 km/h translates to roughly 14 m/s. Thus, reacting and completing a braking manoeuvre even this little bit earlier could potentially help avoid some worst-case scenarios by allowing the vehicle more time to decelerate. While no associated difference in ‘Crash’ or ‘Near Crash’ was detected, this may simply be due to the fact that these outcomes were extremely rare in the first place coupled with the relatively low power of the study. Both before and after training, the mean number of crashes (M_Pre_ = 0.84 vs. M_Pos_ = 0.6) and near crashes (M_Pre_ = 0.53 vs. M_Pos_ = 0.59) for all participants was less than one. This implies that most drivers were capable of responding to all the dangerous events in time, rendering it difficult to demonstrate significant change on these measures. Nevertheless, both the younger and older adult experimental group demonstrated reductions in their mean number of crashes whereas the control group either did not or showed a much smaller difference. Interpreting this result requires caution, however, due to the lack of statistical significance.

Additionally, post-training correlations between driving measures seem to indicate that drivers with greater ‘Distance at Max Brake’ also had lower ‘Max Brake’ and, additionally, that increased ‘Max Brake’ was associated with ‘Crashes’. This—alongside the pattern of correlations between ‘Max Brake’, ‘Crashes’ and other measures of uncontrolled driving—helps reinforce the interpretation that the experimental group exhibited more deliberate and controlled stops.

## Conclusion

To conclude, this study offers preliminary evidence that 3D-MOT training may improve the distance at which participants complete their braking manoeuvres. While we and other researchers have previously demonstrated associations between 3D-MOT and driving ability (Michaels et al., 2017; Woods-Fry et al., 2017), to our knowledge this is the first study suggesting it may be possible to measure the transfer of 3D-MOT training to driving. These results should be interpreted with some caution: sample size concerns due to pandemic-related early termination of the study, the interpretation of trends and the simulated nature of the task limit the generalizability of this study. Additionally, *p*-values were not adjusted for multiple outcome measures given the exploratory nature of the study. As such, the current study is best viewed as a feasibility study as described by the taxonomy from Green et al. (Shawn Green et al., 2019).

The results presented here, while not an unambiguous demonstration of transfer to driving, are a justification for continued research into whether 3D-MOT training improves driving safety. Despite all their advantages, future research of this type should move beyond contrived driving simulator scenarios to truly demonstrate real-world benefits of such training. Looking ahead, such studies could investigate longitudinal driving outcomes following 3D-MOT baseline measures and training much like the ACTIVE study already has for UFOV training.

## Data Availability

The datasets generated and analysed during the current study are available in the Open Science Framework repository, https://doi.org/10.17605/OSF.IO/3V7ZG.
